# Mild cognitive impairment with Lewy bodies: blood perfusion with arterial spin labelling

**DOI:** 10.1007/s00415-020-10271-1

**Published:** 2020-10-21

**Authors:** Michael J. Firbank, John T. O’Brien, Rory Durcan, Louise M. Allan, Sally Barker, Joanna Ciafone, Paul C. Donaghy, Calum A. Hamilton, Sarah Lawley, Jim Lloyd, Gemma Roberts, John-Paul Taylor, Alan J. Thomas

**Affiliations:** 1 Translational and Clinical Research Institute, Newcastle University, Campus for Ageing and Vitality, Newcastle upon Tyne, NE4 5PL UK; 2 Department of Psychiatry, University of Cambridge, Cambridge, UK; 3 College of Medicine and Health, Exeter University, Exeter, UK; 4 Nuclear Medicine Department, Newcastle Upon Tyne Hospitals NHS Foundation Trust, Newcastle upon Tyne, UK

**Keywords:** Mild cognitive impairment, Magnetic resonance imaging, Perfusion imaging, Arterial spin labelling, Lewy body disease

## Abstract

**Objective:**

To use arterial spin labelling to investigate differences in perfusion in mild cognitive impairment with Lewy bodies (MCI-LB) compared to Alzheimer type MCI (MCI-AD) and healthy controls.

**Methods:**

We obtained perfusion images on 32 MCI-LB, 30 MCI-AD and 28 healthy subjects of similar age. Perfusion relative to cerebellum was calculated, and we aimed to examine differences in relative perfusion between MCI-LB and the other groups. This included whole brain voxelwise comparisons, as well as using predefined region-of-interest ratios of medial occipital to medial temporal, and posterior cingulate to precuneus. Differences in occipital perfusion in eyes open vs eyes closed conditions were also examined.

**Results:**

Compared to controls, the MCI-LB showed reduced perfusion in the precuneus, parietal, occipital and fusiform gyrus regions. In our predefined regions, the ratio of perfusion in occipital/medial temporal was significantly lower, and the posterior cingulate/precuneus ratio was significantly higher in MCI-LB compared to controls. Overall, the occipital perfusion was greater in the eyes open vs closed condition, but this did not differ between groups.

**Conclusion:**

We found patterns of altered perfusion in MCI-LB which are similar to those seen in dementia with Lewy bodies, with reduction in posterior parietal and occipital regions, but relatively preserved posterior cingulate.

## Introduction

There is increased recognition that identification and treatment of dementia should be performed as early as possible in the disease process to optimise chances of treatment success, and to be able to offer specific healthcare advice to patients and care givers. As such, there has been a research focus on earlier, prodromal stages, of dementia such as the condition of mild cognitive impairment (MCI), which represents a high risk state for progression to dementia. Whilst most previous work and diagnostic criteria for MCI has focussed around it as a risk state for progression to Alzheimer’s disease, criteria for mild cognitive impairment with Lewy bodies (MCI-LB) have recently been published as part of the recognition that prodromal stages of dementia with Lewy bodies can also be identified [1].

Imaging of brain metabolism, either through FDG-PET, perfusion SPECT or arterial spin labelled MRI (ASL) [2–4] is often done as part of the investigation in dementia patients. Studies with FDG-PET in MCI-LB have found a hypometabolic pattern similar to that seen in dementia with Lewy bodies (DLB) [5], with parieto-occipital hypometabolism [6], and relative preservation of posterior cingulate (cingulate island sign) [7]. Compared with FDG-PET, ASL has the advantage of being cheaper, non-radioactive, and can be done alongside other MR imaging; Dolui [8] compared ASL and FDG in MCI-AD and age matched controls and found similarities between the two techniques, with the hypometabolism patterns in MCI-AD similar to those seen in AD dementia. There have been few studies using perfusion imaging ASL in MCI-LB. Roquet et al. found mild reductions in the temporal and insula gyrus [9], and some studies have compared Parkinson’s disease with and without mild cognitive impairment [10], finding reduced precuneus perfusion. In our previous paper comparing AD and DLB with FDG imaging [11], we found that both the ratio of posterior cingulate cortex (PCC) to precuneus and ratio of medial occipital to medial temporal lobe (MTL) metabolism were useful in distinguishing between the diseases. We aimed to investigate this in MCI using ASL.

In a previous article investigating FDG-PET in early stage PD [12], we found parieto-occipital reductions in PD compared to age matched controls. That study was performed with participants having their eyes open. In healthy subjects with normal vision, occipital metabolism is increased on opening eyes, owing to the increased stimulation of the occipital lobe. We hypothesised that due to changes in their visual system [13], patients with Lewy body disease might have a diminished occipital response to opening their eyes, which might underlie this occipital hypometabolism in the eyes open state. In this current study, we acquired ASL images in both the eyes open and closed conditions to investigate this further in MCI-LB.

In this study, we aimed to investigate blood perfusion measured with ASL in patients with MCI-LB and MCI-AD, in comparison to each other and to healthy subjects of the same age, to verify the extent to which changes seen in DLB are present in MCI-LB and thus to indicate if ASL may be of clinical use in the diagnosis of MCI-LB.

Our hypotheses were:

1. The posterior cingulate/precuneus ratio would be increased in MCI-LB relative to MCI-AD and controls.
2. The medial occipital/MTL ratio would be decreased in MCI-LB relative to MCI-AD and controls.
3. Voxelwise analysis would show decreased perfusion in the occipital and lateral parietal regions in MCI-LB compared with MCI-AD and controls.
4. Blood flow in the occipital lobe would increase in all subjects on eyes open vs closed, but that this would be less marked in MCI-LB.

## Methods

### Participants

This study included participants over the age of 60 with a diagnosis of mild cognitive impairment (MCI) who were recruited from local memory services in the north east of England between April 2016 and Sept 2019. In addition to MCI, participants reported one or more clinical symptoms supportive (but not specific) to a diagnosis of LB disease (e.g. sleep disturbance, mood changes, or autonomic symptoms) or any of the core LB clinical features (visual hallucinations, cognitive fluctuations, REM sleep behaviour disorder, parkinsonism). Following informed consent, participants underwent interview, clinical assessment and neurological examination by a medical doctor (RD, SL).

The MDS Unified Parkinson’s Disease Rating Scale-Motor Examination (UPDRS-III), Epworth Sleepiness Scale (ESS), and Geriatric Depression Scale (GDS) were administered to patients. The Instrumental Activities of Daily Living (IADL) scale, Neuropsychiatric Inventory (NPI), North-East Visual Hallucinations Inventory (NEVHI), Mayo Sleep Questionnaire (MSQ), Dementia Cognitive Fluctuation Scale (DCFS), and Clinician Assessment of Fluctuation (CAF) were administered to informants. Clinical Dementia Rating scale (CDR) and Cumulative Illness Rating Scale for Geriatrics (CIRS-G) were completed on the basis of the clinical history and other research assessments. A detailed neuropsychological evaluation was also carried out similar to that reported previously [14] which included the ACE-R, a 100-point cognitive screening test from which Mini-Mental State Examination (MMSE) score was derived. All participants were offered imaging with 123I-metaiodobenzylguanidine (MIBG) myocardial scintigraphy and 123I-N-3-fluoropropyl-2beta-carbomethoxy-3beta-4-iodophenyl tropane (FP-CIT) SPECT. FP-CIT images were rated as normal/abnormal by an experienced consensus panel blind to clinical information [15]. For MIBG, the heart to mediastinum ratio (HMR) was calculated from manually drawn regions of interest, and scans were classified as normal/abnormal using a predefined HMR cut off of 1.85 based on local control data [16].

Diagnosis of MCI was confirmed by a consensus panel of three experienced old-age psychiatrists according to NIA-AA criteria [17] after reviewing medical records. This was based on evidence of minimal functional impairment and a CDR of 0 or 0.5, and a history of subjective and objective cognitive decline on assessment, originally identified in the health service. We excluded those with dementia or only subjective impairment as well as those with vascular or frontotemporal etiologies, or parkinsonism pre-dating cognitive impairment by more than one year. To determine the aetiology of the MCI, the presence or absence of core Lewy body (LB) symptoms were rated by the panel blind to imaging findings. Core features were determined in accordance with the fourth consensus criteria for DLB [18] which is consistent with the recently published MCI-LB criteria [1].

Participants all had baseline research assessments and most had annual review data available by the time of data locking. Annual review data was used for the consensus panel diagnosis where available. A diagnosis of probable MCI with Lewy bodies (MCI-LB) was given if a patient had two or more core Lewy body symptoms or one core symptom in addition to a positive FP-CIT or MIBG scan. A diagnosis of MCI with probable Alzheimer’s disease (MCI-AD) was given to patients who had no core Lewy body symptoms and negative FP-CIT and MIBG findings. CSF and imaging biomarkers were not used in the diagnosis of MCI-AD, therefore the MCI-AD fulfilled the NIA-AA ‘Core Clinical Criteria’ for MCI-AD.

**Fig. 1:**
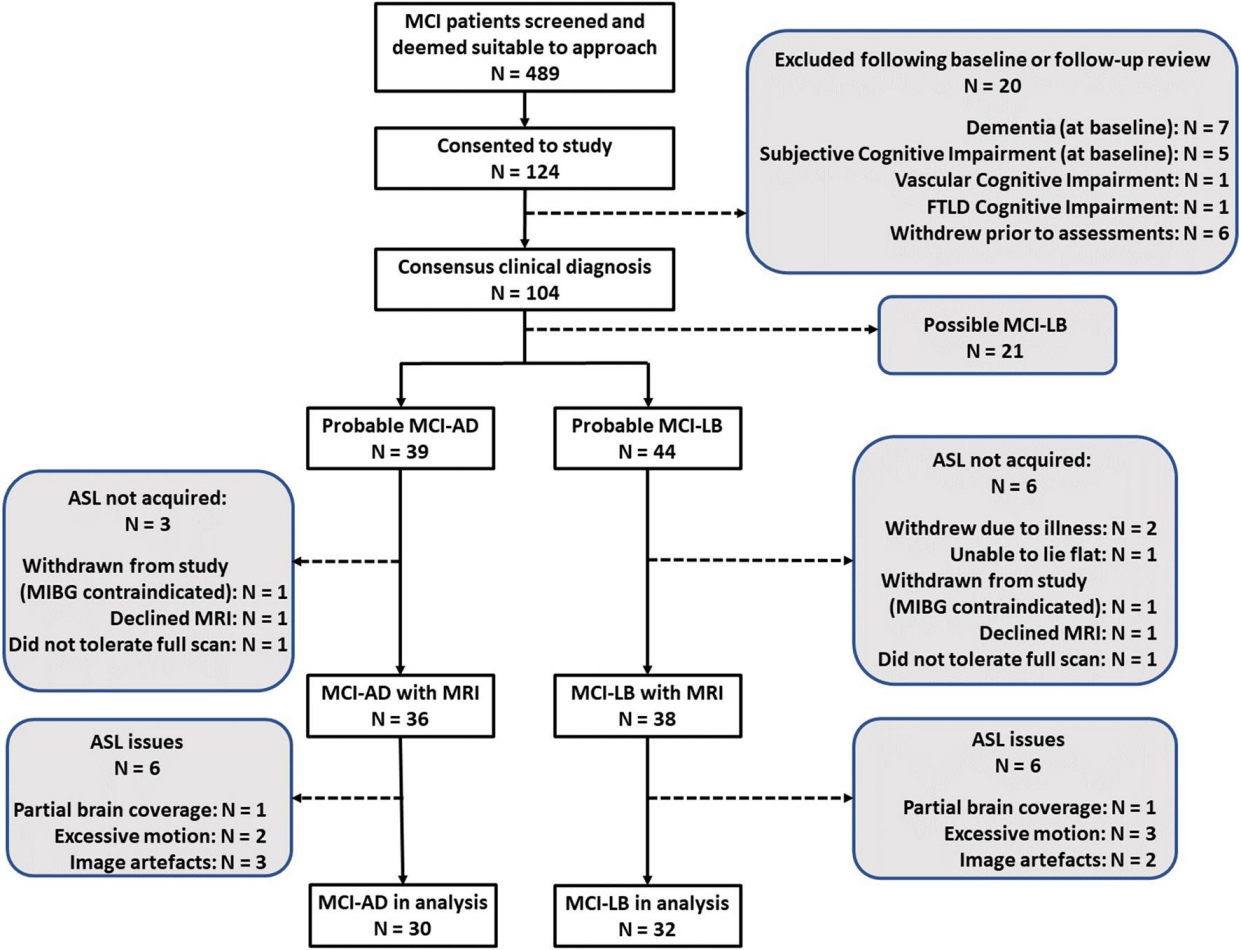
Flow chart showing exclusions and number of MRI scans in the patient groups

According to these criteria, we performed MRI scans on 39 participants diagnosed with probable MCI-LB and 37 diagnosed with MCI-AD. Healthy controls (*N* = 31) were recruited from friends and relatives of the patients and from a local research register and had no history of psychiatric or neurological illness, no evidence of cognitive decline and MR scans within normal limits. We also acquired MRI scans on 18 subjects who had only one Lewy body core feature or positive biomarker at most recent review. These participants were not included in the analysis presented here as our focus was on probable MCI-LB vs MCI-AD. Figure 1 shows the recruitment and exclusion flowchart.

Written informed consent was obtained from all participants prior to study participation and the study was approved by the National Research Ethics Service Committee North East—Newcastle & North Tyneside 2 (Research Ethics Committee Identification Number 15/NE/0420).

### Imaging

Imaging was performed on a 3 T Philips Intera Achieva scanner. Structural images were acquired with a magnetization prepared rapid gradient echo (MPRAGE) sequence, sagittal acquisition, echo time 4.6 ms, repetition time 8.3 ms, inversion time 1250 ms, flip angle = 8°, SENSE factor = 2, in-plane field of view 240 × 240 mm with slice thickness 1.0 mm, yielding a voxel size of 1.0 × 1.0 × 1.0 mm^3^. A FLAIR (fluid attenuated inversion recovery) sequence was also obtained with repetition time 11,000 ms, inversion time 2800 ms, echo time 125 ms voxel size 0.94 × 0.94 mm, 50 slices with thickness 3 mm.

Perfusion scans were acquired in eyes closed then eyes open conditions, each lasting 4 min with a background suppressed pseudo continuous arterial spin labelling (pCASL) with a spin echo- echo planar image (repetition time = 4 s, echo time 13 ms, label duration = 1.65 s, post label delay = 1.6 s, slice thickness = 5 mm). Apart from the comparison of eyes open vs eyes closed, all other analyses were done on the average of both ASL acquisitions.

### Image processing

FLAIR images were co-registered with the T1 weighted structural image, and areas of white matter hyperintensity (WMH) identified using in house developed code based on SPM [19]. The ASL images were converted to relative perfusion maps using the FSL basil tool (https://fsl.fmrib.ox.ac.uk/fsl/fslwiki/BASIL). We used SPM12 (https://www.fil.ion.ucl.ac.uk/spm/) to co-register the ASL images to the structural T1 images, which were normalised to standard space using the Dartel toolbox. The Dartel spatial transformation was applied to the perfusion maps which were written in the standard Montreal Neurological Institute 152 space with a resolution of 1.5 × 1.5 × 1.5 mm. Gaussian smoothing of 8 mm was applied. For region-of-interest analysis, we extracted the mean perfusion values of the medial occipital lobe from the visual area regions V1 and V2 from the anatomy toolbox [20, 21] and used regions of medial temporal lobe, posterior cingulate, precuneus and cerebellum, derived from the AAL atlas [22] as described previously [11]. The mean signal intensity in the cerebellum was used to normalise values to give rCBF in the SPM analyses. The anatomical description of significant clusters was determined utilising the anatomy toolbox.

ASL scans were not obtained on 1 control due to technical problems, 1 MCI-AD and 1 MCI-LB who did not tolerate the whole scan period, two subjects (1 MCI-AD, 1 MCI-LB) were excluded due to incomplete scan coverage of the brain. We excluded those with > 2 mm motion (1 control, 2 MCI-AD, 3 MCI-LB). We also visually inspected all scans (blind to group) and excluded a further 1 control, 3 MCI-AD, 2 MCI-LB due to poor quality images including those with low signal to noise, unexpected asymmetries, and abnormally high signal in the main blood vessels.

### Statistics

For image voxelwise comparisons, we used the general linear model in SPM, thresholded voxelwise at *p* < 0.001 uncorrected, and then clusters which were significant after family wise error correction (*p* < 0.05) were reported. R version 3.6.3 was used for all other statistical analysis. ANOVA was used to compare groups, followed (where significant) by Tukey HSD test to determine between group differences. *T* tests were done without assuming equal variance. Chi square or Fisher’s exact tests were done to compare categorical variables. Correlations with clinical variables were done with Spearman’s rho. For WMH volumes, we calculated the log of the ratio of WMH to total brain volume to render the data more normally distributed. Table 1 gives the mean volume in mm^3^ for ease of interpretation, but the t-test was done on the logged values.

**Table 1:** Subject demographics for those included in the analysis

	Control [*N* = 28]	MCI-AD [*N* = 30]	MCI-LB [*N* = 32]	Comparison statistics [MCI-AD vs -LB for clinical scores]
Age (SD) [range]	73.4 (7.0) [61–89]	75.8 (7.7) [62–87]	73.2 (5.6) [60–83]	*F*_2,87_ = 1.41 *p* = 0.25
Female	9/28 (32%)	16/30 (53%)	3/32 (9%)	*X*^2^ = 13.98 *p* < 0.001
Years in education (SD) [range]	14.4 (4.0) [8–24]	12.7 (3.3) [10–20]	12.3 (2.9) [10–21]	*F*_2,84_ = 2.87 *p* = 0.062
UPDRS Motor score	5.8 (4.4)	13.2 (11.2)	22.8 (14.5)	*t*_57.9_ = − 2.93 p = 0.005
MMSE	28.4 (1.1)	26.7 (2.1)	26.5 (2.4)	*t*_59.7_ = 0.34 p = 0.73
ACE Memory	22.5 (3.1)	18.0 (4.7)	20.0 (4.6)	*t*_59.5_ = − 1.76 p = 0.084
ACE visuospatial	15.6 (0.7)	14.9 (1.3)	14.0 (2.0)	*t*_53.8_ = 2.01 p = 0.049
ACE total	92.6 (4.1)	82.4 (8.7)	84.2 (9.2)	*t*_60.0_ = − 0.80 *p* = 0.43
Epworth total	4.7 (3.0)	5.0 (4.6)	8.3 (4.4)	*t*_59.3_ = − 2.88 *p* = 0.006
GDS	1.3 (1.9)	3.3 (2.4)	5.1 (4.2)	*t*_50.0_ = − 2.07 *p* = 0.044
IADL		7.3 (1.3)	6.4 (1.4)	*t*_50.0_ = 2.33 *p* = 0.024
CDR total	0.0 (0.0)	0.5 (0.0)	0.5 (0.1)	*t*_31.0_ = 1.79 *p* = 0.083
NPI total		7.3 (8.8)	14.1 (11.3)	*t*_50.8_ = − 2.46 p = 0.017
Cholinesterase Inhibitors	0/27 (0%)	6/28 (21%)	14/31 (45%)	*X*^2^ = 2.71 *p* = 0.099
Antiparkinsonian drugs	0/27 (0%)	0/28 (0%)	4/31 (13%)	FET, *p* = 0.11
Antidepressant drugs	3/27 (11%)	4/28 (14%)	13/31 (42%)	FET, *p* = 0.024
Antipsychotic drugs	0/27 (0%)	0/28 (0%)	0/31 (0%)	FET, *p* = 1.00
Anxiolytic drugs	1/27 (4%)	0/28 (0%)	1/31 (3%)	FET, *p* = 1.00
Total WMH volume (mm^3^) (SD) [Range]	4.56 (4.67) [0.3–15.4]	10.18 (9.93) [0.2–31.2]	8.71 (8.12) [0.2–39.4]	*t*_58.3_ = 0.30 *p* = 0.76

Apart from age, sex and education, all reported statistical comparisons are between MCI-AD and MCI-LB

Values are mean (SD) or *N* (%)

*UPDRS* United Parkinson’s Disease Rating Scale (part III), *MMSE* mini mental state exam, *ACE *Addenbrooke’s cognitive exam, *Epworth* Epworth Sleepiness Scale, *GDS* Geriatric Depression Scale, *IADL* Instrumental Activities of Daily Living, *NPI* Neuropsychiatric Inventory, *CDR* Clinical dementia rating scale, *RBD* REM sleep behaviour disorder. *FET* Fisher’s exact test. *WMH* white matter hyperintensity. The statistics on the WMH were performed on the log values

## Results

There were 32 MCI-LB, 30 MCI-AD and 28 controls with ASL perfusion scans included in the analysis. Table 1 shows the demographics of these subjects. In keeping with the disease profile, most of the MCI-LB subjects were male. Results from these participants have been previously published [23], including further details of the cognitive and clinical characteristics of the patients.

Table 2 shows the mean values of perfusion for the PCC/precuneus ratio. This was highest in the MCI-LB group, which was significantly different to the control group, but there were no significant differences between the two MCI groups. Table 2 also shows the ratio of medial occipital/medial temporal lobe perfusion. This was lowest in MCI-LB, again being significantly different in comparison to controls, but not MCI-AD. As an indication of the tagging efficiency variablity, the measured signal in the occipital lobe in the perfusion weighted images (ie without normalisation by the cerebellum) was: Control 16.5 (SD 5.5), MCI-AD 18.4 (SD 6.1), MCI-LB 14.4 (SD 4.5) arbitrary units.

**Table 2:** Perfusion ratio in posterior cingulate/precuneus (cingulate island) and medial occipital/medial temporal lobe

	Control (*N* = 28)	MCI-AD (*N* = 30)	MCI-LB (*N* = 32)	ANOVA statistics
Posterior cingulate/precuneus	0.99 (0.11)	1.02 (0.12)	1.08 (0.15)	*F*_2,87_ = 4.26 *p* = 0.017 (MCI-LB v control *p* = 0.014 MCI-LB v MCI-AD *p* = 0.17)
Medial occipital/MTL	1.22 (0.22)	1.20 (0.24)	1.08 (0.20)	*F*_2,87_ = 3.81 *p* = 0.026 (MCI-LB v control *p* = 0.042 MCI-LB v MCI-AD *p* = 0.064)

Tukey post hoc comparisons are shown in brackets

**Fig. 2:**
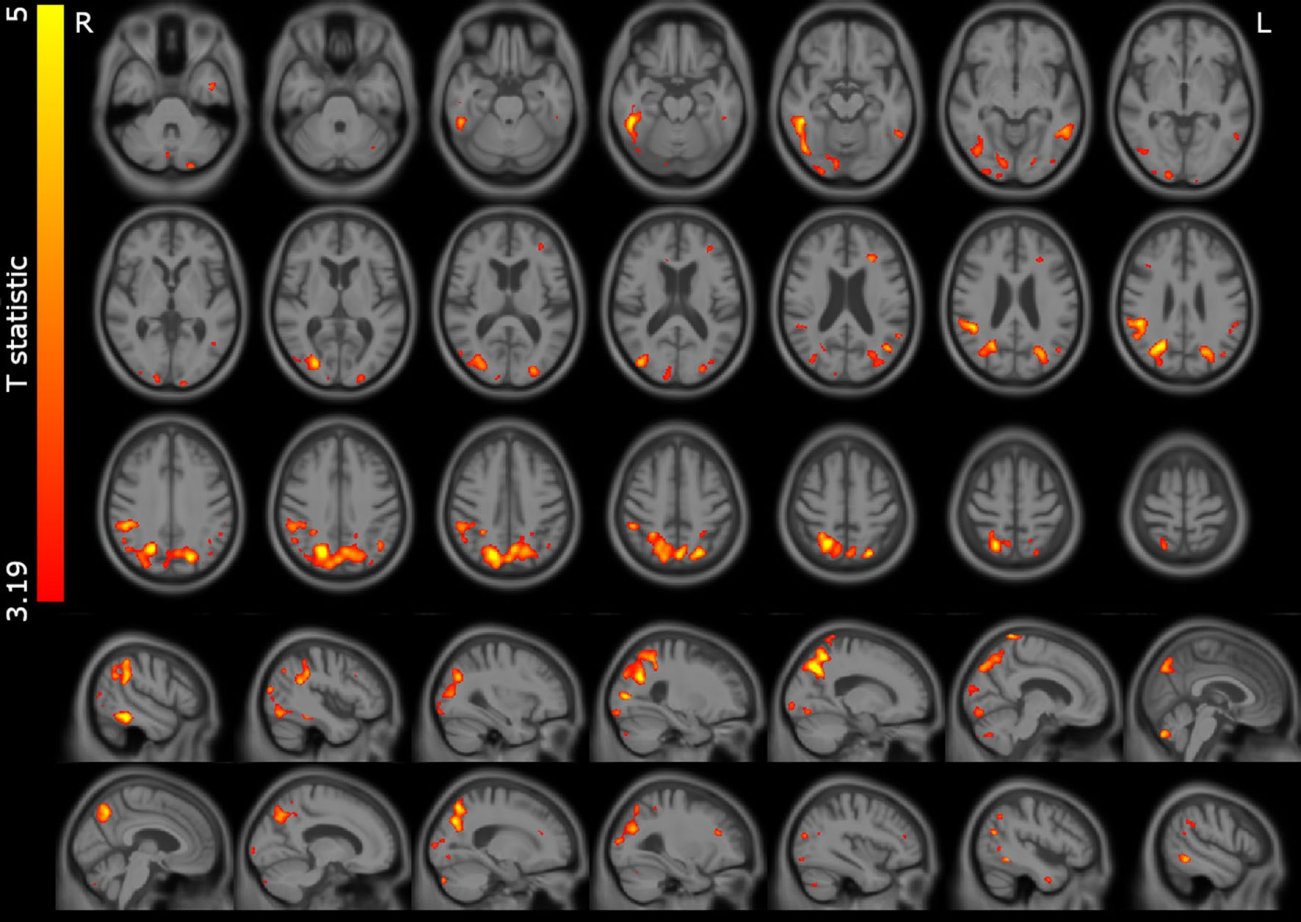
Significant perfusion reductions in MCI-LB relative to the control group. T statistic map overlaid on study specific average structural scan. Results thresholded at *p* < 0.001 voxelwise

For the voxelwise comparisons (Fig. 2, Table 3), the MCI-LB compared with the control group had significantly lower perfusion in the precuneus and superior parietal, parietal-occipital, and fusiform gyrus occipito-temporal areas. We extracted the mean signal from the three significant clusters (precuneus and superior parietal, inferior parietal, and occipito-temporal) to investigate correlations with clinical scales in the MCI-LB group.

**Table 3:** Significant clusters for the comparison MCI-LB < control from the voxelwise analysis

*p* value (FWE corrected)	Cluster size (mm^3^)	Representative coordinates in MNI space (*x*, *y*, *z* mm)	Location description
< 0.001	35,519	20, − 68, 40	Precuneus and superior parietal
		21, − 58, 56	
		26, − 64, 28	
0.001	5734	51, − 39, − 20	Inferior occipital/temporal lobe, including fusiform gyrus
		45, − 69, − 14	
		38, − 58, − 9	
< 0.001	7303	48, − 39, 32	Right inferior parietal
		45, − 44, 26	
		50, − 39, 50	

A threshold of *p* < 0.001 uncorrected for multiple comparisons was applied voxelwise, and significant clusters (*p* < 0.05) family wise error (FWE) corrected for multiple comparisons are reported

*MNI* Montreal Neurological Institute space

There were no significant clusters in the voxelwise comparison between MCI-AD and either control or MCI-LB groups.

**Fig. 3:**
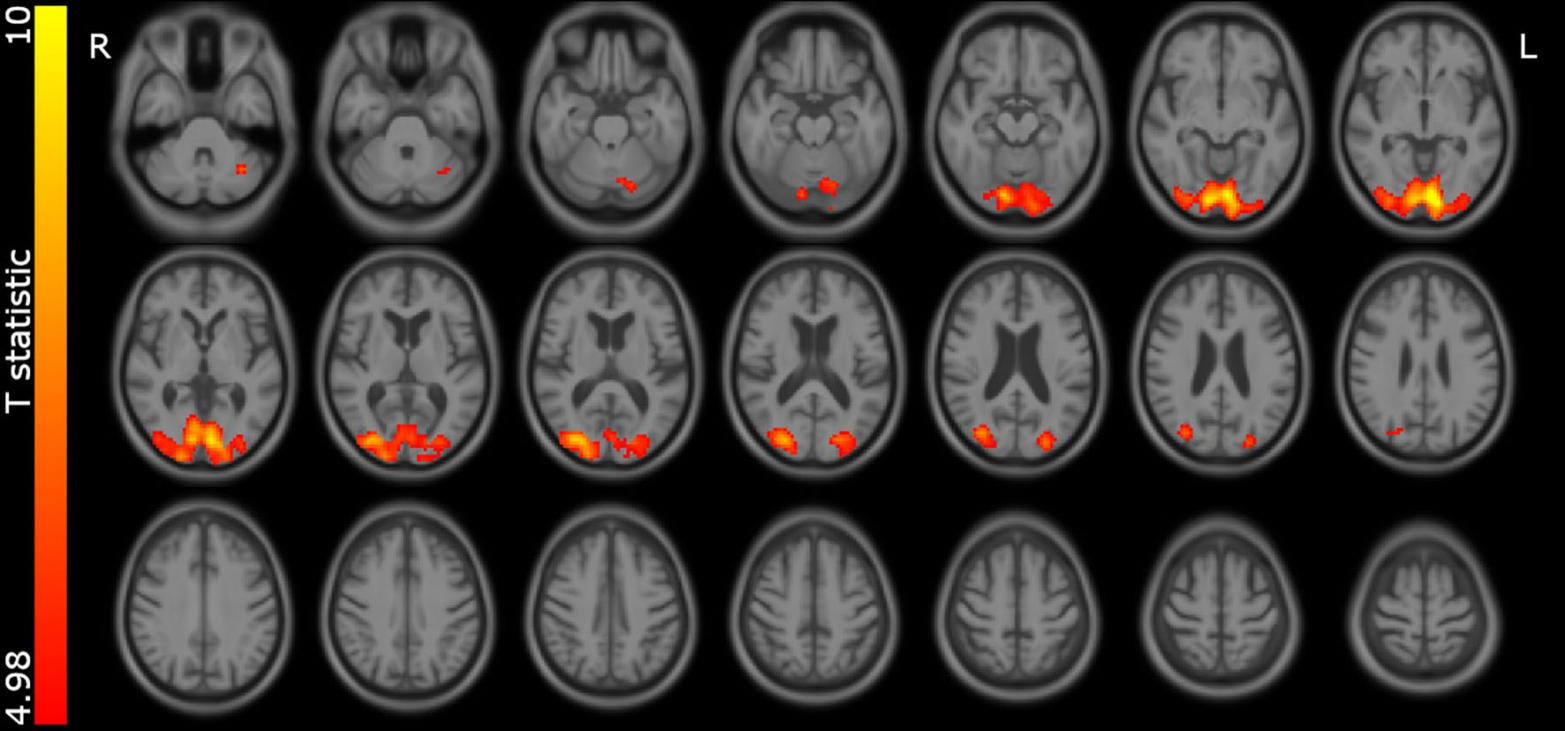
Areas where perfusion is greater during eyes open vs closed in the entire group. Statistic map thresholded at *p* = 0.05 voxelwise family wise error corrected. Overlaid on study specific average structural scan

In the comparison of eyes open vs closed, as expected there was significantly higher perfusion in the primary visual cortex of the occipital lobe during the eyes open vs eyes closed period (Fig. 3). However, as can be seen in Table 4, there were no significant group differences in the change in perfusion to eyes opening. Table 4 also shows the occipital perfusion in the eyes closed state, showing a significant reduction in MCI-LB compared to the control group.

**Table 4:** Percentage change in perfusion in primary visual areas on eyes opening compared between groups, and relative CBF in the eyes closed condition between groups

	Control (*N* = 28)	MCI-AD (*N* = 30)	MCI-LB (*N* = 32)	ANOVA statistics
CBF % change in medial occipital on opening eyes	10.30 (7.52)	7.04 (14.20)	9.11 (10.40)	*F*_2,87_ = 0.65 *p* = 0.53
CBF eyes closed medial occipital /cerebellum	3.33 (0.38)	3.15 (0.48)	3.01 (0.45)	*F*_2,87_ = 4.05 *p* = 0.021 (MCI-LB v control *p* = 0.016 MCI-LB v MCI-AD *p* = 0.40)

Tukey post hoc comparisons are shown in brackets

**Fig. 4:**
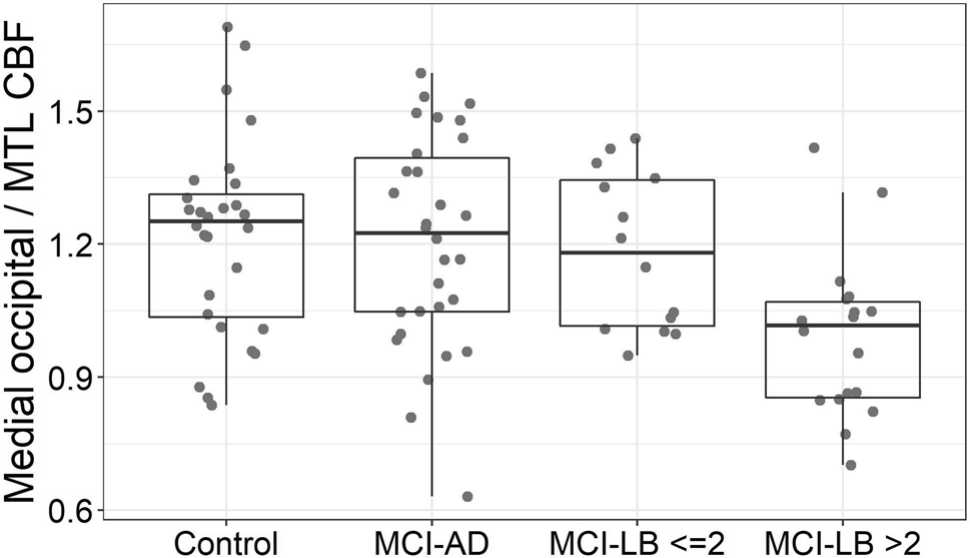
Medial occipital/medial temporal lobe perfusion ratio in the groups, with the probable MCI-LB group split by the number of core diagnostic features

To investigate the extent to which the perfusion changes in the MCI-LB group were more marked in those subjects with a more robust LB disease phenotype, we compared those with two or fewer diagnostic features at baseline (including clinical features and abnormal MIBG or FP-CIT) against those with more than 2 features. As shown in Fig. 4, compared to those with two or fewer diagnostic features, the MCI-LB participants with more diagnostic features had a significantly lower medial occipital/MTL perfusion ratio (0.992 SD 0.18 vs 1.180 SD 0.18; *t* = 3.01; *p* = 0.005). The PCC/ precuneus ratio was also greater (though not significantly) in this group (1.13 SD 0.17 vs 1.03 SD 0.11; *t* = 2.00; *p* = 0.054). Although perfusion relative to cerebellum was lower in all the spm clusters in the MCI-LB group with more diagnostic features, this was not significant: superior parietal (2.78 SD 0.65 vs 3.2 SD 0.76; *t* = 1.67; *p* = 0.1), inferior parietal (3.38 SD 0.59 vs 3.49 SD 0,70; *t* = 0.47, *p* = 0.6), occipito-temporal (2.26 SD 0.40 vs 2.4 SD 0.60; *t* = 0.86; *p* = 0.4).

To see whether loss of cognitive abilities typical of LB disease were associated with perfusion loss, we also investigated associations between perfusion in these regions in the MCI-LB participants and the ACE Total score, as well as ACE visuospatial and memory composite score (Table 5) representing domains frequently affected in LBD and AD respectively. The parietal cluster perfusion correlated significantly with ACE visuospatial score, and tended to significance with ACE total and memory score; however, these were not significant after controlling for multiple comparisons.

**Table 5:** Spearman correlations in MCI-LB patients of perfusion in the spm clusters and the two ROI ratios against ACE total score, ACE visuospatial and ACE memory subscore

	ACE Total	ACE visuospatial	ACE memory
Parietal/cerebellum	rho = 0.34; *p* = 0.055	rho = 0.39; *p* = 0.026*	rho = 0.33; *p* = 0.064
Occipo-Temporal/cerebellum	rho = 0.01; *p* = 0.97	rho = 0.28; *p* = 0.12	rho = 0.07; p = 0.70
Inferior Parietal/cerebellum	rho = − 0.06; *p* = 0.74	rho = 0.08; p = 0.65	rho = − 0.06; *p* = 0.75
PCC/precuneus	rho = 0.00; *p* = 0.98	rho = − 0.09; *p* = 0.63	rho = − 0.02; *p* = 0.90
Medial occipital/MTL	rho = 0.17; *p* = 0.36	rho = 0.26; *p* = 0.16	rho = 0.11; *p* = 0.54

^*^p < 0.05

## Discussion

We found patterns of altered perfusion in MCI-LB which are similar to those seen in DLB, with reduction in posterior parietal and occipital regions and a significantly lower medial occipital/MTL perfusion ratio compared with healthy controls, and evidence of the cingulate island sign, although this did not distinguish MCI-LB from MCI-AD.

The overall pattern of hypoperfusion in MCI-LB is very similar to that seen in DLB [3, 11, 24] albeit less pronounced, with posterior reductions in perfusion, including the precuneus, inferior parietal, occipital lobe, and inferior temporal lobe, and relative sparing of the posterior cingulate gyrus (see Fig. 2). This implies that the typical perfusion deficits in DLB are present from an early stage, and encourages the use of ASL or FDG-PET to identify those at risk of DLB. We saw some evidence (Table 4) that the perfusion in the precuneus and superior parietal lobe was reduced in those with worse cognition. These areas are affected in both AD and DLB [2, 11, 24], and correlations between cognition and glucose metabolism in this region have likewise been reported in PD [12]. Reduced perfusion in the precuneus and adjacent parietal areas thus seems to relate to cognitive decline in general, rather than being specific to any particular disease.

The relative preservation of the posterior cingulate in DLB is well established, being visible using both FDG and ASL [3]. A study by Iizuka et al. [25] concluded that the cingulate island sign changed with LB disease progression in an inverted U shaped fashion, being most marked in those with an MMSE of 22 compared to those with higher or lower MMSE. In keeping with this, we found the ratio of posterior cingulate to precuneus was increased overall in MCI-LB, tending to be more so in those with more diagnostic features of LB disease. This finding supports the use of this ratio (or a visual rating scale assessment [26]) to identify those with more typical LB features. However, a recent review in MCI-LB [5] suggested that reduced posterior cingulate metabolism can occur in those who progress to DLB, and further work is needed to validate the use of posterior cingulate preservation as a predictor of LB disease.

In our previous research in DLB with FDG-PET, we suggested the use of the medial occipital to medial temporal lobe ratio to distinguish AD from DLB. Recent research in RBD (a frequent prodromal stage of Parkinson’s disease and DLB) has identified a pattern with (amongst other regions), relatively increased hippocampus, and decreased occipital glucose metabolism [27, 28] with similarities to the alterations seen in DLB and PD [29] indicating that the pattern may be typical of LB disease. We found reductions in the occipital/MTL ratio in MCI-LB with more LB features, compared to controls, which in combination with the previous research suggests that this may be a useful measurement for identification of those with Lewy body disease.

We did not see any significant perfusion changes in MCI-AD compared to either the control group, or MCI-LB. Although several other reports have found perfusion differences in MCI-AD with ASL [8, 30, 31], others have not [32]. The extent to which hypoperfusion is demonstrated likely depends on disease severity, as well as the group homogeneity. Since all our MCI participants had to have some LB related symptoms (e.g. anosmia or visual disturbance), it may be that our MCI-AD group, whilst meeting standard diagnostic criteria, is more inclusive than those typically studied, and thus the perfusion values more varied than in a highly selected cohort. It could also be that the relatively small sample size of the study gave insufficient power to detect a difference.

As expected, we observed greater perfusion in the primary visual cortex in the eyes open vs eyes closed condition in all groups. In our paper [12] looking at glucose metabolism in early stage PD we had observed a reduced metabolism in the occipital lobe. The imaging in that study was performed with FDG injected in the eyes open state. We had hypothesised that in PD, increase in glucose metabolism on eyes opening might be diminished due to reduced engagement with the visual environment. In the results presented here, the change in perfusion between eyes open and closed did not differ between groups. Furthermore, occipital hypoperfusion in MCI-LB relative to the control group was observed in the eyes closed perfusion data (see Table 5). This implies that the occipital hypometabolism and hypoperfusion seen in Lewy body disease is not related to retinal changes which are seen in LB disease [13]. Since there also appears to be relatively little pathology seen in the primary visual cortex in Lewy body disease [33, 34], this suggests that the metabolism changes are secondary to altered connectivity with other brain regions.

Strengths of our study include the prospective design and consensus clinical assessment, along with the use of two imaging biomarkers (FP-CIT and MIBG), leading to strong confidence in the patient groups. We did not have specific biomarkers for AD, however, our MCI-AD group had negative FP-CIT and MIBG scans, both of which, individually, have good specificity for the diagnosis of DLB vs AD [38, 39] which gives strong support to the MCI-AD diagnosis. Limitations of our study include that the ASL technique is sensitive to head movement, and we had to exclude a number of participants due to poor quality CBF images. Other studies have also excluded similar proportions of scans due to poor image quality [3, 8]. We also used relative rather than absolute CBF, as we did not acquire a suitable reference image, however Dolui et al. [8] found no difference in overall discriminatory ability between relative and absolute CBF. Due to changes in the vascular system, it could be that both MCI-AD and MCI-LB have altered arterial transit time relative to controls [35]. The ASL imaging in this study was performed with a single fixed delay between the magnetic tagging of incoming blood and the acquisition of the image. This potentially leads to underestimation of perfusion if the delay is not well matched to the arterial transit time, however investigating this fully requires a long ASL protocol. It is possible that BOLD (blood oxygenation level dependent) changes may have influenced the ASL signal on eyes opening. However, we used a short echo time and background suppression, which have been found to minimize the influence of BOLD on pCASL [36]. ASL relies on subtraction of two sets of images (control and tag), with the tag images having inflowing blood magnetically labelled. It is possible that mismatch between the control and tag images could lead to magnetisation transfer effects [37] and thus macromolecule differences (eg aggregated pathological proteins) may potentially have contributed to the signal.

Overall, we found perfusion changes in MCI-LB to be similar to the hypoperfusion/hypometabolism patterns seen in DLB but consistent with the earlier stage of disease these appear to be less prominent. Thus we found few significant differences between MCI-LB and MCI-AD perfusion patterns, suggesting that ASL may be of limited utility diagnostically in mild cognitive impairment, though the medial occipital/MTL ratio might be of use and needs further exploration.
